# A Vade-Mecum of Morbid Anatomy, Medical and Chirurgical; with Pathological Observations and Symptoms

**Published:** 1831

**Authors:** 


A Vade-Mecum of Morbid Anatomy, Medical and Chirurgical; with Pathological Observations and Symptoms. Illustrated by upwards of 250 Drawings. Large 8vo. pp. 51. Burgess and Hill, London, 1831.

To insist on the advantages resulting from the cultivation of morbid anatomy would be a work of supererogation at the present time, when they are felt and appreciated by almost every member of the profession. Neither is it germain to our purpose to point out the ill-consequences which are likely to result from an over-weening fondness for such researches, nor to show how a great good may be converted into a great evil. Many able works upon morbid anatomy, and delineations of structural alterations have been published, and years must elapse before we shall be enabled to excel Cruvelhier and Baillie in this department. But a manual or vade-mecum has hitherto been wanting, and, considering the rapidity with which manuals are produced and the avidity with which they are purchased, it is somewhat surprising that morbid anatomy has not hitherto possessed one. The deficiency is now supplied, and the anonymous author of the present work deserves encouragement for the laudable attempt which he has made to accommodate the junior portion of our brethren. It contains observations on, with illustrations of the changes of structure found in the brain, thoracic, abdominal, and pelvic viscera, and of the organs of generation in both sexes. Appended to each plate is a brief description of the symptoms and morbid anatomy of the disease represented. The plates are lithographed, the accompanying descriptions succinct and tolerably satisfactory. We have said already that the author deserves commendation, both for the conception and execution of his undertaking; but we should deceive our readers and himself if we declared that it was not susceptible of improvement. An extract or two will enable the profession to judge for itself. We will select, at hazard, the disorganizations of the fauces and œsophagus.

"PLATE XIII.

"Sketches of chronic inflammation of the fauces, enlarged, indurated, and ulcerated tonsils.

SYMP. Tickling in the throat, attended with great heat and a peppery taste in the mouth, cough, expectoration of mucus, sometimes blood; the tonsils are enlarged, and of a dark red colour. The uvula is soft and papulous, as if composed of a clot of blood. The capillary veins are distended, and the soft vascular network spread over the palate gives it the appearance of a well injected tissue of blood vessels.

MORBID ANAT. *Cynanche Maligna*—The fauces are inflamed, suppurated, and gangrenous; and the trachea and larynx are likewise in a state of inflammation, and lined with a viscid fetid matter.

PLATES XIV. XV.

These plates represent acute and chronic inflammation of the œsophagus and its effects.

SYMP. *Inflammation of the Œsophagus*—Burning sensation on taking food, pain in the neck, particularly so in the side, which is increased on pressure, dryness of the fauces, regurgitation of the food through the nostrils.

*Ulceration*—An uneasy sensation in some part of the œsophagus, which is aggravated after eating, or after drinking ardent spirits; a lancinating or burning sensation in one or more parts of the tube; slight cough, cardialgia, repeated efforts to clear the throat of a tough dirty-coloured matter.

SYMP.—*Stricture of the Œsophagus*—Dyspnœa, difficulty in swallowing, nausea, vomiting, eructation, sense of hunger, bowels constipated, dejected and pallid countenance, cramp and spasms, both at the seat of stricture and in the stomach.

MORBID ANAT. The mucous membrane is generally found pulpy and red, and its capillary vessels are very distinctly seen loaded with blood; sometimes it is lined with a false membrane. Ulcers of the œsophagus are generally surrounded with indurated and everted edges; the erosion sometimes is very deep, sometimes superficial." 14.

We have only space for one other quotation.

SYMP.—*Acute Muco-Enteritis*—Compressed state of the abdomen, pain in the region of the navel; nausea, vomiting, slight tormina; jelly-like evacuations; tongue covered with a whitish fur, red tip and edges; pulse soft and compressible.

*Chronic, with Ulceration*—Cough; rigors with alternate flushes of heat; jelly evacuations, mixed with pus or blood; tongue covered with a dirty yellowish fur, red tip and edges, pulse soft and compressible, pain on pressure just above the pubis; belly becomes flatter, the face more sunk, and the cheeks and eyes more hollow.

*Mucous Memb. of the large Intestines*—Tormina, tenesmus; scanty evacuations of feculent matter, blended at times with mucus, pus, or blood; pulse quick and full, tongue white, with slight red tip and edges.

*Stricture of the Gut*—Pain and sense of weight in the loins, which sometimes extends to the rectum and thighs; obstruction to free evacuation, with a thready and compressed appearance of the feculent matter; pallid countenance; if of long standing, great emaciation.

*Cancer of the Rectum*—Severe pain, darting through the pelvis to the bladder and the groin. The countenance is of a sallow leaden colour.

MORBID ANAT. The parts are redder than usual, and the vessels are seen to course in an arborescent manner; there is an œdematous and puffy state of the part, which is produced by an infiltration of fluid into the cellular connecting membrane. Lymph is sometimes found adhering to its surface. The redness in acute inflammation has no line of demarcation, but vanishes gradually from a dark red to a faint flesh colour.

*Chronic*—The mucous tissue is thickened, attenuated, softened, indurated, or ulcerated; and the colour of the parts varies considerably, being at times blue, red, green, yellow, white, or black.

Encephaloid tumours differ in their structure from fungus hæmatodes, and likewise in their manner of destroying parts. These tumours are found to be of a medullary consistency, and protected by one or more tunics, according to the part affected. The capsules are bountifully supplied with arteries and nerves, and the venous capillary vessels are seen to form complete masses of vessels on their external surface. Some of the vessels enter into the substance of the tumour. Sometimes the medullary matter occupies one cell only; sometimes six or eight, so as to form one tumour, hence the lobulated appearance. The intervening cellular tissue is matted together by chronic inflammation, so as not to be separated without the greatest difficulty. In the substance of these tumours there is most commonly found a small quantity of blood.

*Fungus Hæmatodes*—has no proper capsule, and possesses the peculiar power of changing every portion of the human frame to its own nature; and this occurs without that thickening or hardening of the cellular tissue which generally accompanies chronic inflammation.

In ulceration, the capillary vessels of the venous system form a perfect net-work; and these minute vessels press into larger branches of the venous system: it is to the varicose and partial lesion of these vessels, that the edges and inferior surface of the ulceration owe their dark ruby colour. If the ulceration be extensive, the destruction of veins is very great, and hemorrhage is the consequence." 40.

The foregoing specimens of the Vade Mecum will show pretty fairly its style and character. The plates are rather too small to convey very accurate perceptions to those who are not frequently in the habit of studying from Nature; and the detail of symptoms is too brief to afford very substantial information. Yet it would have been difficult to have avoided these errors, without, at the same time, falling into others of an opposite description. On the whole, we are disposed to speak favourably of the volume, and to recommend it to our junior brethren.

